# Book review of *Introduction to U.S. Health Policy: The Organization, Financing and Delivery of Health Care in America *by Donald A. Barr

**DOI:** 10.1186/1747-5341-3-9

**Published:** 2008-03-03

**Authors:** Audrey R Chapman

**Affiliations:** 1 Division of Medical Humanities, Health Law and Ethics, Department of Community Medicine and Health Care, University of Connecticut School of Medicine, Farmington, CT 06030, USA

## Abstract

Donald A. Barr's *Introduction to U.S. Health Policy: The Organization, Financing, and Delivery of Health Care in America *(second edition, 2007) offers a lucid and informative overview of the U.S. health system and the dilemmas policy makers currently face. Barr has provided a balanced introduction to the way health care is organized, financed, and delivered in the United States. The thirteen chapters of the book are quite comprehensive in the topics they cover. Even those knowledgeable about the U.S. health care system are likely to find much to stimulate their thinking in the text. The book can also appropriately serve as a basic text for a health policy course or in the medical or nursing school curriculum.

## Review

Currently there is widespread dissatisfaction with the health system in the United States. Health care reform has once again emerged as a priority domestic policy issues at the national level, and it is likely to play an important role in the forthcoming U.S. presidential election. Following the example of Massachusetts, several states are also considering health care reform initiatives. Notably, even organizations like the American Medical Association and major corporations, which once ardently opposed comprehensive health care reform, are advocating for major structural changes in the U.S. health care system.

Several trends account for much of the current momentum toward health care reform. The first is escalating health costs. While in 1970 people in the United States spent an average of $341 per person on all types of health care, by 2003 this figure had risen to about $5,670 per person, a figure which is more than 50 percent higher than any other industrialized country and 140 percent above the average for OECD (Organization for Economic Cooperation and Development) countries [1]. Although other developed countries also face the pressure of rising health care costs, primarily from covering the cost of new and more expensive technologies and pharmaceuticals and meeting the health care needs of aging populations, the U.S. problem is more severe [2].

A second issue is gaps in coverage and access to care. The U.S. is the only industrialized democracy that does not recognize a right to health care and/or provide universal health insurance for its citizens. Despite escalating health costs and the highest per capita investment in health care in the world, one-sixth of the U.S. population lacks health insurance, and the numbers are rising every year. Disturbingly, the absence of health care insurance falls disproportionately on vulnerable groups, specifically low-income Americans and ethnic minorities, particularly blacks, Hispanics, and native Americans. As might be anticipated, lack of health insurance for these groups often translates into the failure to obtain timely and appropriate health care.^a^

Yet another factor accounting for the support for health care reform is that the system of employer-based insurance on which the U.S. uniquely relies is unraveling. While the costs of health insurance benefits were once modest, today health coverage for a family of four is estimated to be more than $10,000 [3]. Given the financial pressures, many employers are reducing health insurance benefits, transferring more costs onto employees, or dropping insurance for some or all employees. In 2005, nearly eight in ten uninsured people, equivalent to more than one-fifth of the adult work force, came from families with at least one full time worker [4].

In addition, there are problems with the quality of health care. Indicators on health performance show that the U.S. health system compares unfavorably with other advanced and even some middle income countries in terms of life expectancy, infant mortality rates (with the U.S. having the lowest of 23 industrialized countries), and avoidance of preventable mortality [5]. A 2000 WHO study ranked the overall performance of the U.S. health system as 37^th ^of 191 countries [6].

A variety of approaches to health care reform have been put forward, but with quite different goals and emphases. Several, including a Canadian style single-payer approach and various proposals to impose individual and or/corporate mandates for health insurance, seek to find ways to cover some or all of the 47 million U.S. citizens who are currently without health insurance. In contrast, the emphasis in other plans, as for example health savings accounts, is on preserving or increasing choice and reducing health-related costs. It is difficult, however, to assess the proposals being put forward to reform the U.S. health system without understanding the factors leading to the problems noted above and the reasons that major health care reform initiatives were unsuccessful in the past.

Donald A. Barr's *Introduction to U.S. Health Policy: The Organization, Financing, and Delivery of Health Care in America *(second edition, 2007) can serve as such a needed resource. The book offers a lucid and informative overview of the U.S. health system and the dilemmas policy makers currently face. Barr, who is trained both as a physician and a sociologist and holds an appointment as an Associate Professor of Sociology and Human Biology at Stanford University, has provided a balanced and inclusive introduction to the way health care is organized, financed, and delivered in the United States. The volume provides a helpful introduction to the U.S. health care system for anyone wishing to understand how the U.S. can simultaneously have the best and worst of health care systems among industrialized countries. Even those knowledgeable about the U.S. health care system are likely to find much to stimulate their thinking in the text. The book can also appropriately serve as a basic text for a health policy course or in the medical or nursing school curriculum.

The thirteen chapters of the book are quite comprehensive in the topics they cover. The first chapter opens with a brief historical description of the unique history of health care in the U.S. and the policy decisions that have shaped the current system. The chapter provides data about the costs of healthcare, and the burdens that these increases impose on both the public and the private sectors. It also briefly compares the U.S. with other developed countries in terms of the amount we spend on health care and the overall health status of our population. The introduction has sections on concerns about the quality of health care and additionally introduces the problem of the growing number of the uninsured, another characteristic unique to the U.S. health care system among industrialized democratic countries.

The second chapter describes many of the institutional norms, cultural values, and expectations that have shaped the unique character of the U.S. health care system. It highlights American "exceptionalism" by briefly comparing the U.S. and Canadian health systems. One particularly insightful section of this chapter examines how fundamental cultural and value differences and between the U.S. and Canada are reflected in their respective health care systems.

The third chapter addresses the professional structure of U.S. health care. It covers the history of medical education and the medical profession as well providing a brief analysis of the development of the nursing profession. It examines the structure of hospitals and other types of specialized referral centers. This chapter documents the unusually dominant role physicians and the American Medical Association have played in shaping the character of the medical system. Barr also mentions how the medical profession organized to block previous efforts to reform the system.

The next few chapters offer an overview of health care structures. Chapter 4 focuses on health insurance and the development of health maintenance organizations. Chapters 5 and 6 describe the history of Medicare and Medicaid and explore the policy questions confronting these two major government health care programs. Chapter 7 considers the increasing role of for-profit health care in the delivery of health care and some of the consequences for the health care system.

Chapter 8 focuses on pharmaceutical policy and the rising cost of prescription drugs examining the way the U.S. health system organizes, pays for, and delivers pharmaceutical products to patients. Some of the topics included in this discussion of what has become one of the central issues of U.S. health policy are pharmaceutical research and the development of "me-too" drugs; the marketing of new drugs to physicians; the lack of meaningful oversight of physicians' relationships with the pharmaceutical industry; past efforts to control pharmaceutical costs; and the 2006 Medicare prescription drug benefit.

Long-term care is the subject of chapter 9. The topics discussed include the growing need for long-term care among frail elderly people, home health care, hospice care, and life-care communities as an alternative to long-term care.

Chapters 10 and 11 return to the problem of access to health care. Chapter 10 deals with the uninsured, and chapter 11 addresses factors other than health insurance that impede access to health care. Barr points out that the U.S. is alone among developed countries in maintaining national policies that exclude segments of the population from health insurance coverage and explains how and why this has occurred. He analyzes who the uninsured are and projects what the future numbers are likely to be unless there are new initiatives to provide coverage. Non-insurance barriers that Barr identifies include out-of-pocket expenses, the inadequacy of Medicaid coverage, disparities in treatments offered to members of different racial groups, and the organizational complexity of the health care system.

The final two chapters explore topics related to health care reform. Chapter 12 focuses on key policy issues for deciding on the direction. According to Barr, to be successful in restructuring the U.S. system of health care, we will need to deal simultaneously with the three problems that lie at the center of U.S. health policy: constraining the cost of care, maintaining and improvement the quality of care, and increasing access to care. He also considers whether rationing health care is inevitable and under what kinds of conditions it might be acceptable. Chapter 13 concludes with a brief consideration of some of the options for health care reform.

One of the strengths of this book is that Barr consistently places the health care system in a broad social and cultural context. He draws on theories from fields as diverse as economics, sociology, and organizational behavior to assess the broad social forces that coalesce to create the structure of U.S. health care and the problems inherent in it. His discussion of the cultural expectations and institutions that drive the U.S. health care system is particularly insightful.

Barr contrasts the primacy accorded to the rights of the individual in the U.S. with the greater emphasis on the common good in other countries, like Canada, that provide universal health care. He points out that this difference in disposition has resulted in very different organizing principles for the Canadian and U.S. health care systems. In Canada health care is a basic right; the power of the medical profession is limited by its social obligation; health care is organized through a single-payer system; and there is one standard of health care for all Canadians. In addition, Canadian political culture recognizes the need for limits in health care expenditures and accepts the appropriateness of the system allocating scarce health care resources to those in greatest need, measured in terms of the risk to their life or health.

In contrast, Barr shows that the principles around which the U.S. health care system has come to be organized are quite different and contribute to current problems. In the U.S., health care is considered to be a market commodity to be distributed according to the ability to pay rather than a basic right; power over the organization and delivery of health care has historically been concentrated in the medical profession without a strong countervailing tradition of social obligation; even as its contribution to financing the health system has grown, government has historically had relatively little role in guiding the system of health care; and there is no uniform standard of care as in Canada, but instead, the quality of care received often reflects the ability to pay. In addition, Barr points out that the U.S. culture lacks a sense of the need for limits in expenditures on health care. Thus there is an always rising public demand for access to new pharmaceuticals and technologies, even when there is no evidence that they are necessarily more effective.

Given the importance of health care reform, it is disappointing that Barr does not give this issue more attention. His analysis clearly underscores that the current U.S. system of health care is not sustainable. Barr shows that there are simply too many areas of potential crisis and financial instability for the system to survive without substantial reform. Nevertheless, he offers a superficial discussion of the options. A short final chapter of only 13 pages provides a very brief description of four alternative approaches and with a few phrases highlights their potential strengths and weaknesses. It does not assess the prospects for adoption of any of these models or alternative plans. This is an important issue, especially in light of the multiple past attempts to reform the U.S. health care system. Perhaps the third edition of the book will cover this issue in greater depth or, even better, be able to provide an analysis of successful future health care reforms.

## Endnote

**a**. See the Kaiser Family Foundation website particularly "Key Facts: Race, Ethnicity and Medical Care 2007 Update," "The Uninsured: A Primer," and "How Trends in the Health Care System Affect Low-Income Adults: Identifying Access Problems and Financial Burdens" [7].

## About the author

Dr. Chapman is the Joseph M Healey, Jr. Professor of Medical Humanities and Ethics at the University of Connecticut Medical School. She is the author, coauthor, or editor of sixteen books and numerous articles and reports dealing with ethical, human rights, theological, and intellectual property issues related to health, genetic developments, and pharmaceuticals. She also has published works on economic, social and cultural rights; health care reform; transitional justice; reconciliation; and development issues.

## Competing interests

The authors declare that they have no competing interests.
